# The relevance of Spearman's *g* for epilepsy

**DOI:** 10.1093/braincomms/fcae176

**Published:** 2024-06-12

**Authors:** Aaron F Struck, Camille Garcia-Ramos, Veena A Nair, Vivek Prabhakaran, Kevin Dabbs, Lisa L Conant, Jeffrey R Binder, David Loring, Mary Meyerand, Bruce P Hermann

**Affiliations:** Department of Neurology, University of Wisconsin–Madison, Madison, WI 53726, USA; Department of Neurology, William S. Middleton Veterans Administration Hospital, Madison, WI 53705, USA; Department of Neurology, University of Wisconsin–Madison, Madison, WI 53726, USA; Department of Radiology, University of Wisconsin–Madison, Madison, WI 53726, USA; Department of Radiology, University of Wisconsin–Madison, Madison, WI 53726, USA; Department of Neurology, University of Wisconsin–Madison, Madison, WI 53726, USA; Department of Neurology, Medical College of Wisconsin, Milwaukee, WI 53226, USA; Department of Neurology, Medical College of Wisconsin, Milwaukee, WI 53226, USA; Department of Neurology and Pediatrics, Emory University, Atlanta, GA 30322, USA; Department of Medical Physics, Wisconsin-Madison, Madison, WI 53726, USA; Department of Neurology, University of Wisconsin–Madison, Madison, WI 53726, USA

**Keywords:** neuropsychology, brain morphology, graph theory, resting-state fMRI

## Abstract

Whilst the concept of a general mental factor known as ‘*g*’ has been of longstanding interest, for unknown reasons, it has never been interrogated in epilepsy despite the 100+ year empirical history of the neuropsychology of epilepsy. This investigation seeks to identify *g* within a comprehensive neuropsychological data set and compare participants with temporal lobe epilepsy to controls, characterize the discriminatory power of *g* compared with domain-specific cognitive metrics, explore the association of *g* with clinical epilepsy and sociodemographic variables and identify the structural and network properties associated with *g* in epilepsy. Participants included 110 temporal lobe epilepsy patients and 79 healthy controls between the ages of 19 and 60. Participants underwent neuropsychological assessment, clinical interview and structural and functional imaging. Cognitive data were subjected to factor analysis to identify *g* and compare the group of patients with control participants. The relative power of *g* compared with domain-specific tests was interrogated, clinical and sociodemographic variables were examined for their relationship with *g*, and structural and functional images were assessed using traditional regional volumetrics, cortical surface features and network analytics. Findings indicate (i) significantly (*P* < 0.005) lower *g* in patients compared with controls; (ii) *g* is at least as powerful as individual cognitive domain-specific metrics and other analytic approaches to discriminating patients from control participants; (iii) lower *g* was associated with earlier age of onset and medication use, greater number of antiseizure medications and longer epilepsy duration (*P*s < 0.04); and lower parental and personal education and greater neighbourhood deprivation (*P*s < 0.012); and (iv) amongst patients, lower *g* was linked to decreased total intracranial volume (*P* = 0.019), age and intracranial volume adjusted total tissue volume (*P* = 0.019) and age and intracranial volume adjusted total corpus callosum volume (*P* = 0.012)—particularly posterior, mid-posterior and anterior (*P*s < 0.022) regions. Cortical vertex analyses showed lower *g* to be associated specifically with decreased gyrification in bilateral medial orbitofrontal regions. Network analysis of resting-state data with focus on the participation coefficient showed *g* to be associated with the superior parietal network. Spearman's *g* is reduced in patients, has considerable discriminatory power compared with domain-specific metrics and is linked to a multiplex of factors related to *brain* (size, connectivity and frontoparietal networks), *environment* (familial and personal education and neighbourhood disadvantage) and *disease* (epilepsy onset, treatment and duration). Greater attention to contemporary models of human cognition is warranted in order to advance the neuropsychology of epilepsy.

## Teaser

Struck *et al*. address Spearman's classic ‘*g*’ factor in participants with temporal lobe epilepsy (*n* = 110) and controls (*n* = 79) and show *g* is lower in epilepsy and linked to *brain* (size, connectivity and frontoparietal networks), *environment* (familial and personal education and neighbourhood disadvantage) and *disease* (epilepsy onset, treatment and duration).

## Graphical Abstract

**Graphical Abstract:**
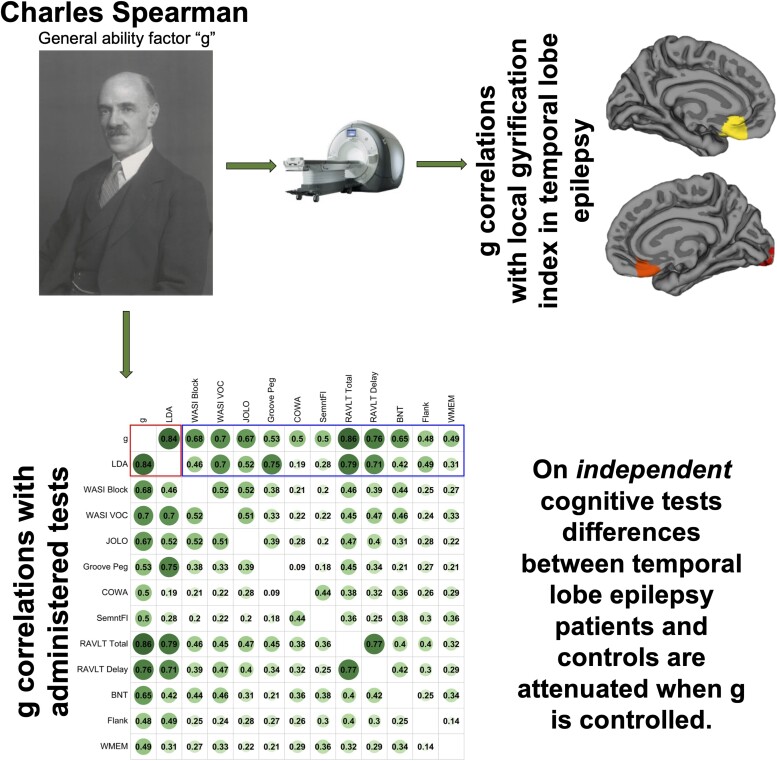

## Introduction

The administration of intelligence tests to children and adults with epilepsy has been a longstanding tradition in the neuropsychology of epilepsy. The first report of intelligence testing appeared in 1912 when, soon after the Binet–Simon was translated to English, it was administered to residents of an epilepsy colony in New Jersey,^1^ one of many such centres where early cognitive research in epilepsy took place.^2-4^ The Binet–Simon and its subsequent modifications (e.g. Stanford–Binet), in concert with development of numerous additional tests of intelligence (e.g. Weschler–Bellevue, Progressive Matrices), their later revisions, downward extensions (e.g. Weschler Intelligence Scale for Children) and translations, ultimately formed an extensive international literature addressing the relationship between epilepsy and intelligence test scores developed through population- and community-based investigations and innumerable clinical studies.^5,6^

These various tests, heterogeneous in content, all purported to assess the underlying construct of intelligence and were used to examine the course of intellectual development in youth with epilepsy, follow its trajectory in midlife and track its vulnerability with increasing chronicity of epilepsy and advancing age. Intelligence tests have also been used to interrogate the effects of various treatments (e.g. medications, surgery and diet), predict clinical outcomes, clarify the impact of diverse clinical characteristics (e.g. seizure frequency, status epilepticus and frequency of generalized seizures),^5-7^ compare the relative standing of diverse epilepsy syndromes^8^ and address potential benefits linked to higher intelligence test scores (i.e. cognitive reserve).^9^ Research examining epilepsy–intelligence test relationships continues to this day,^10-12^ spanning a course of approximately 110 years.

But with few exceptions (e.g. Halstead's Biological Intelligence Tests,^13^ Woodcock–Johnson Tests of Cognitive Abilities,^14^ Kaufman Assessment Battery for Children, Differential Abilities Scale^15^ and Luria Nebraska Neuropsychological Battery),^16^ the most frequently used tests in epilepsy research were not initially constructed on the basis of conceptual or theoretical models of intelligence. Rather, they emanated from tests and metrics derived to identify schoolchildren at risk of school failure,^17^ assess immigrants at Ellis Island who were inadept at English but needed to demonstrate mental capacity for entry to America (so-called performance tests),^18^ classify persons for military and institutional purposes^19^ and in the worst instances carried out by the eugenics movement, to assist in the identification of ‘defectives’ for the purposes of segregation and prevention of reproduction.^20^ Developments in the fields of psychometrics and individual differences occurred over the decades to be sure, but epilepsy research remained largely agnostic to the identification or development of a meaningful biological or conceptual characterization of intelligence, instead simply relying on the availability of existing measures purporting to assess ‘intelligence’.

As clinical and research interest came to focus more on specific domains of cognitive ability in epilepsy such as memory, executive function and language, the unique role of intelligence tests, other than as an overall marker of ability level, arguably diminished compared with its prominent position in the earlier literature.^5^ Consensus has not been achieved regarding how best to integrate intelligence test scores into domain-specific investigations of epilepsy. Because of shared variance between intelligence tests and measures of specific cognitive abilities (e.g. memory), differences between epilepsy and control groups can be weakened or even negated when intelligence is covaried, a point both argued and criticized.^9,21^

This intercorrelation of diverse measures of cognitive ability amongst themselves, as well as with measures of intelligence, was noted early on by Spearman who coined the term ‘positive manifold’ to refer to these broad consistent patterns of association.^22^ Spearman argued that a general ability factor, called ‘*g*’, represented a major underlying dimension of general cognitive ability.^22,23^ Research has demonstrated that *g* can be identified in diverse cognitive data sets globally, is evident in cognitive metrics from animal species, with long-term predictive significance for important life outcomes, and demonstrated hereditability and underlying neuroimaging signatures.^24-26^ Yet to date, we are unaware of any investigation of *g* in epilepsy despite its long history in the general field of individual differences and the extended history of intelligence test research in epilepsy. But interest is growing in the applicability of empirically based models of intelligence including *g* and specific cognitive abilities to epilepsy, for example by using the Cattel–Horn–Carroll model.^27-30^

In that context, the current research has four aims: (i) identify and then contrast *g* in persons with epilepsy to healthy control participants; (ii) characterize the relative effect size of *g* compared with specific cognitive abilities in epilepsy versus control group comparisons and identify the unique variance in specific cognitive domains after controlling for *g*'s contribution; (iii) relate *g* to clinical epilepsy characteristics and sociodemographic factors; and (iv) identify the structural and functional imaging correlates of *g* in persons with epilepsy. These tasks are undertaken in a multicentre investigation of persons with temporal lobe epilepsy (TLE) and controls with comprehensive accompanying cognitive, clinical and neuroimaging information.

## Materials and methods

### Participants

Participants include 110 TLE patients and 79 healthy controls from the Epilepsy Connectome Project (Table 1),^31,32^ a two-site research project involving the Medical College of Wisconsin and the University of Wisconsin–Madison. TLE participants were between the ages of 19 and 60 and spoke English fluently, had no medical contraindications to MRI and with estimated short form *Wechsler Abbreviated Scale of Intelligence* (WASI)-2 Full Scale Intelligence Quotient at or above 70. Two patients had intelligence quotient under 70 but with significant verbal versus performance asymmetry with one score intact and they were retained. TLE diagnosis and side of seizure onset were determined by a board-certified neurologist with expertise in epileptology following the criteria defined by the International League Against Epilepsy. TLE participants should meet two or more of the following: (i) clinical semiology consistent with seizures of temporal lobe origin; (ii) proof of either Temporal Intermittent Rhythmic Delta Activity or temporal lobe epileptiform discharges in an EEG, (iii) onset of temporal lobe seizures captured on video EEG monitoring; or (iv) MRI indication of mesial temporal sclerosis or hippocampal atrophy. Exclusion criteria included (i) seizures caused by lesions other than mesial temporal sclerosis and (2) seizures originated by an active infectious/autoimmune/inflammatory conditions.

**Table 1:** Clinical and sociodemographic characteristics

	TLE (*N* = 110)	Controls (*N* = 79)
Age	39.8 (11.7)	34.3 (10.7)
Gender (F/M)	67/43	45/34
Education	14.7 (2.7)	15.7 (2.6)
Mother education	13.5 (2.7)	14.6 (2.7)
Father education	13.8 (2.9)	14.7 (2.8)
Age of onset	23.0 (13.6)	
Duration	16.7 (14.2)	
Estimated lifetime GTC seizures	13.0 (22.7)	
Number of ASMs	1.83 (0.93)	
Laterality (EEG) (L/R/B/U)	57/24/9/20	

ASMs, antiseizure medications; GTC, generalized tonic-clonic.

Controls participants were healthy adults between 18 and 60 years of age. Exclusion criteria included left handedness, English as a second language, history of any learning disability, brain injury or affliction, substance abuse, use of vasoactive medications, major psychiatric illness like depression, bipolar disorder or schizophrenia and medical contraindications to MRI. All participants provided written informed consent according to the Declaration of Helsinki, and the study was reviewed and approved by the Institutional Review Board at Medical College of Wisconsin, and all experiments were performed in accordance with relevant guidelines and regulations.

### Neuropsychology

The healthy control and epilepsy participants underwent neuropsychological evaluation including nine metrics from a test of intelligence (WASI-2 Vocabulary and Block Design subtests),^33^ verbal learning and memory (Rey auditory verbal learning test) including total words learned across trials and delayed recall,^34^ object naming (Boston Naming Test),^35^ letter fluency (Controlled Oral Word Association Test),^36,37^ semantic fluency (Animal Naming),^36,38^ spatial orientation (Judgement of Line Orientation)^39^ and speeded fine motor dexterity (Grooved Pegboard, dominant hand).^40^ In addition, selected cognitive tests from the National Institutes of Health (NIH) Toolbox-Cognitive Battery were administered,^41^ including Pattern Comparison Processing Speed,^42,43^ Dimensional Change Card Sort, List Sorting Working Memory, Flanker Inhibitory Control and Attention (Flank), Picture Vocabulary (VOCAB), Oral Reading Recognition (ORAL) and Picture Sequence Memory tests. Measures used in the factor analysis included the nine traditional cognitive test metrics along with the NIH ToolBox Flanker and List Sorting Working Memory tests, included to assess executive function. The remaining NIH ToolBox measures were used in a secondary analysis to address TLE versus control group differences without and then with *g* as a covariate.

### Clinical and sociodemographic predictors of *g*

Participants underwent structured health, educational and social interviews that included for epilepsy participants, medical record review to characterize the aetiology, course, clinical characteristics and treatment of their epilepsy. Familial and social variables included formal years of education of parents and the Area Disadvantage Index (ADI) for each participant, which summarizes 17 US Census poverty, education, housing and employment indicators, characterizing neighbourhood disadvantage in specific census-based regions.^44^ Participants were categorized into advantaged (ADI Quintiles 1 and 2) and disadvantaged (ADI Quintiles 4 and 5) groups rather than individual quintiles given sample size distributions.

### Neuroimaging

#### Image acquisition

Per protocol, 55 controls and all TLE patients underwent neuroimaging. MRI was performed on 3T General Electric MR750 scanners using a Nova 32-channel head coil at both institutions. T_1_-weighted structural images were acquired using MPRAGE (reduced magnetization prepared gradient echo) sequence, with the following parameters: repetition time/echo time = 604 ms/2.516 ms, inversion time = 1060.0 ms, flip angle = 8°, field of view = 25.6 cm and voxel size = 0.8 mm isotropic. Four 5-min resting-state functional MRI (rs-fMRI) images were acquired over two sessions using whole-brain simultaneous multi-slice, gradient echo planer imaging (8 bands, 72 slices, repetition time/echo time = 802 ms/33.5 ms, flip angle = 50°, matrix = 104 × 104, field of view = 20.8 cm and voxel size = 2.0 mm isotropic) and a 32-channel receive coil were concatenated.^45^ Participants were asked to focus on a white cross at the centre of a black screen during the scans for better reliability.^46^

#### MRI pre-processing

Neuroimages were processed using the Human Connectome Project minimal processing pipelines,^47^ mainly based on FreeSurfer v5.3 and Functional MRI of the brain Software Library,^48,49^ and the functional part mainly based on Analysis of Functional Neuroimages.^50^ General details on the Human Connectome Project processing pipelines can be found in Glasser *et al*.^47^ and specific details in our previous work.^32,51^ Pairwise Pearson correlations between time series across the brain were calculated and Fisher's *z* transformed for the generation of connectivity matrices used for graph theory (GT) analyses.

#### Matrices creation for GT analyses

Resting-state functional MRI matrices based on temporal correlations between nodes were constructed for 50 healthy controls and 101 TLE participants, and GT metrics were then calculated. Nodes consisted of 360 cortical regions based on the Glasser Parcellation,^52^ and 19 subcortical areas from FreeSurfer automatic parcellation, for a total of 379 nodes, can be found in Garcia-Ramos *et al*.^51^ Once the matrices were created, we used a combination of proportional thresholding with the Minimum Spanning Tree as its backbone (to avoid *disconnected* nodes) as a thresholding method. Therefore, a density value in this publication refers to such combination (see Garcia-Ramos *et al*.^53^ for details). GT measures were calculated for each participant with Matlab-based Brain Connectivity Toolbox, and afterwards averages were used for analyses.

### Analyses

#### Neuropsychology

##### *g* in TLE and control groups (Aim 1)

Spearman's *g* was derived using exploratory factor analysis on 11 neuropsychological tests administered to all participants (epilepsy and controls combined). The Kaiser–Meyer–Olkin factor was checked for adequacy. Kaiser–Meyer–Olkin factor greater than 0.8 is considered meritorious.^54^ Individual measures of sampling adequacy were also collected for each item. Bartlett correlation tests were performed to determine whether the correlations were sufficient for factor analysis. To determine the optimal number of factors, the minimum average partial test was performed looking at both the 1976 and 2000 criteria. Gradient projection rotations were used for factor rotation with oblique rotation. Exploratory factor analysis was used for minimum average partial test.

##### Explanatory power of *g* compared with domain-specific tests (Aim 2)

Linear discriminant analysis (LDA) was used to create a linear combination of cognitive variables that optimally separated the epilepsy and control groups. The intention was to compare the LDA latent variable with *g* to determine whether the variance between groups could be explained by differences in *g*. LDA was performed with ‘MASS’ package in *R*.^55^ In addition, the NIH ToolBox cognitive measures not incorporated in the construction of *g* were compared without and then with *g* as a covariate to further understand its impact on epilepsy versus control comparisons.

#### Clinical and sociodemographic predictors of *g*

##### Relationship of *g* to demographic, clinical epilepsy, familial and social factors (Aim 3)

The relationship of *g* to categorical variables (e.g. gender, laterality and ADI) was explored by *χ*^2^, whilst continuous variables (e.g. age, education, age of onset and duration of epilepsy) were examined by Pearson or Spearman correlation depending on the distributional characteristics of the predictor variables.

#### Neuroimaging

Three sets of analyses examining *g* with structural and functional measures were conducted (Aim 4).

#### Volumetric analyses

The association of *g* with hypothesis-driven volumetric measures of interest was undertaken including with total intracranial volume (ICV), total tissue volume, total left and right grey matter volumes, corpus callosum volume and volumes of thalamus and hippocampi.

#### Cortical morphology

FreeSurfer's Query, Design, Execute, Contrast was used to perform surface-based analysis for cortical volume [age, gender, ICV covariates], thickness (age and gender covariates), area (age and gender covariates) and Local Gyrification Index (age and gender covariates) measurements. Local Gyrification Index is calculated as the ratio of the area of cortex hidden within the sulcal folds of the pial surface to the area of cortex on the external visible cortex of the smoothed surface and was calculated to compare cortical complexity between groups. Each subject's surface measures were mapped to the atlas surface ‘fsaverage’ to allow between-subject comparisons. Surface data were smoothed with a 10 mm full width at half maximum filter. A Monte Carlo simulation was implemented to correct for multiple comparisons (cluster forming threshold set to *P* = 0.05).

#### GT measures and analysis

Properties of global clustering, global integration and network configuration were explored by calculating normalized transitivity, normalized global efficiency and modularity index, respectively. Transitivity is the fraction of ‘triangles’ (closed connection between three nodes) to ‘triplets’ (connections between three nodes) in the network.^56,57^ This measure reflects how clustered or segregated a network is. Global efficiency is defined as the average of the inverse of the shortest paths in the network.^58^ Therefore, high global efficiency represents the integration of communication within the network. Properties of clustering and efficiency in complex networks part from their comparison with the same properties in random networks. Therefore, in order to explore global clustering and efficiency, the same measures were calculated in random matrices with the same number of nodes and degree distribution than the pertaining graphs and were used to normalize them; therefore, measures were divided by their random counterparts. The modularity index (*Q*) exposes the capacity of the network to be subdivided into highly connected subnetworks or modules that often contribute to the same underlying neurological processes.^59,60^ In a highly modular network, nodes within the same modules may be involved in the same specialized process. In this study, *Q* was estimated using the modularity Louvain algorithm.^59^ These global measures are calculated at different density levels to assess the consistency of results regardless of the number of connections in the matrices.

In addition to these global measures, the participation coefficient (PC) was calculated, which represents a nodal measure that explores the associations of a node with respect to a given modular segmentation.^61^ Here, we explored PC based on the 22 Glasser parcellations as well as an additional parcellation containing 17 subcortical structures and bilateral cerebellum (see Supplementary Table 1). Glasser parcellations are based on marked differences in cortical architecture, function, connectivity and topography in a group of 210 healthy young adults from the Human Connectome Project.^52^ These include visual-, motor- and auditory-related regions, amongst others. Calculating PC based on these parcellations (i.e. networks of regions) would help elucidate how similarly segregated these are in TLE since PC facilitates determination of whether connections are mainly contained to one parcellation/module or are shared across different ones. A value close to 1 means that *connections* from a node are not exclusive to one module. Therefore, the lower the PC, the more contained or exclusive to one module the connections of a node are. Given that the choice of density value could render variable results, we calculated PC at 20, 30 and 40% density in order to verify consistency of outcomes. A threshold of 40% was chosen to be officially presented in the manuscript given that it might be the most representative of the actual brain functional associations without being too dense and containing negative associations. Results for 20 and 30% can be found in Supplementary Figs. 1 and 2, respectively.

## Results

### Participants

Clinical and sociodemographic characteristics of the sample are presented in Table 1. The TLE group was modestly but significantly older than controls and had more females. These variables were controlled for in the analyses to be described.

### Neuropsychology

#### Status of *g* in TLE and control groups (Aim 1)

With Kaiser–Meyer–Olkin analysis the overall measure of sampling adequacy (MSA) was 0.84 with individual tests having MSA > 0.80. All MSA are considered ‘meritorious’ per Kaiser's original description. Further, Bartlett test had a *P*-value < 0.001 also suggestive of adequacy of the factor analysis. The number of factors was determined using the minimum average partial test. The optimal number of factors was 1 in both the 1976 and revised 2000 criteria. The root mean square of residual was 0.08. Tucker–Lewis Index of factoring reliability was 0.77. Correlation of regression scores with factors was 0.93. Multiple *R*^2^ of scores with factors was 0.87.

#### Explanatory power of *g* and domain-specific tests (Aim 2)

**Figure 1:**
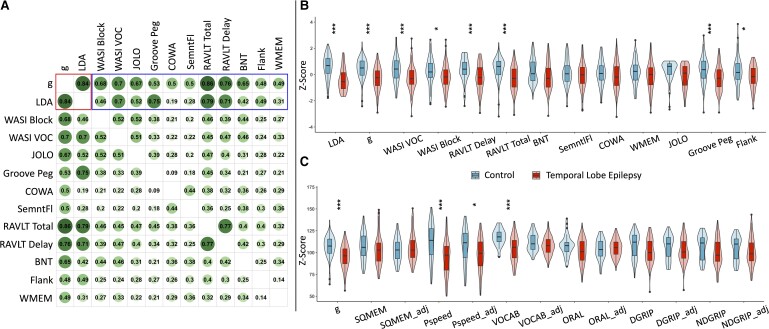
**Correlation plot and cognitive testing between groups.** (**A**) Correlation plot between cognitive tests used to generate *g* and the results of the LDA—a machine learning tool to find a linear combination of variables that maximal separates TLE from controls. The numbers are Pearson correlations. In the left box are the correlation between *g* and LDA results. In the right box are the weights of each cognitive test for *g* and LDA. (**B**) Differences in cognitive tests between TLE and controls. (**C**) Differences in cognitive tests between TLE and controls from the NIH cognitive toolbox before and after adjustment for *g* factor; *g* is also present for comparison. Permutation tests equivalent to a Student's *t*-test were performed: *P < 0.05, **P < 0.01 and ***P < 0.001. LDA, linear discriminant analysis; WASI, Wechsler Abbreviated Scale of Intelligence; VOC, vocabulary; JOLO, Judgement of Line Orientation; SemntlFl, semantic fluency; RAVLT, Rey auditory verbal learning test; BNT, Boston Naming Test; Flank, Flanker inhibitory control and attention; WMEM, working memory; Peg, pegboard; SQMEM, sequence memory; Pspeed, processing speed; VOCAB, picture vocabulary; ORAL, oral reading recognition; DGRIP, grip strength of dominant hand; NDGRIP, grip strength of non-dominant hand; adj, adjusted for *g*; NIH, National Institutes of Health; TLE, temporal lobe epilepsy.

There were significant differences between TLE and controls across several metrics including *g*, vocabulary and block design subtests (WASI-2), total verbal learning and delayed recall (Rey auditory verbal learning test), speeded fine motor dexterity (Grooved Pegboard) and selective attention and inhibition (Flanker; Fig. 1B). Whilst *g* was significantly lower in the epilepsy group, there was expected heterogeneity in performance in both groups with overlapping distributions, which is depicted in Supplementary Fig. 3. The effect sizes for *g*, LDA and individual test metrics in epilepsy versus control comparisons are shown in Table 2. As can be appreciated, verbal learning/memory and speeded fine motor dexterity have the largest effect sizes. Figure 1A shows the associations between the 11 core administered tests (11 × 11 = 121) as well as their association with *g* and LDA (13 × 13 = 169). Apparent is the high degree of intercorrelation across all test metrics (positive manifold), the very significant association of *g* with all individual test metrics and the high correlation between *g* and LDA at 0.86. The LDA factor was more highly correlated with *g* than any individual test with the next highest being total verbal learning (Rey auditory verbal learning test) at 0.79, demonstrating that *g* was more discriminatory than any individual test metric.

**Table 2:** Effect sizes for *g*, LDA and individual test metrics

	TLE	Control	*P*-value	Cohen *d*
*g*	−0.307 ± 0.876	0.427 ± 0.846	0.000	0.850
LDA	−0.407 ± 0.841	0.567 ± 0.929	0.000	1.109
Block design (WASI-2)	−0.185 ± 0.938	0.257 ± 1.033	0.035	0.452
Vocabulary (WASI-2)	−0.269 ± 0.913	0.375 ± 1.000	0.000	0.677
Line orientation (JOLO)	−0.210 ± 1.110	0.292 ± 0.735	0.009	0.516
Motor speed (Pegboard)	−0.280 ± 0.917	0.390 ± 0.985	0.000	0.709
Letter fluency (COWA)	−0.079 ± 1.042	0.110 ± 0.934	1.000	0.190
Semantic fluency (animals)	−0.107 ± 1.017	0.148 ± 0.963	1.000	0.256
Verbal learning (AVLT-I)	−0.310 ± 0.955	0.432 ± 0.901	0.000	0.796
Delayed recall (AVLT-DR)	−0.277 ± 0.999	0.386 ± 0.870	0.000	0.700
Naming (BNT)	−0.165 ± 1.012	0.230 ± 0.942	0.097	0.401
Attention/inhibition (Flanker)	−0.189 ± 0.872	0.263 ± 1.108	0.028	0.463
Working memory	−0.125 ± 1.045	0.174 ± 0.912	0.555	0.301

BNT, Boston Naming Test; COWA, Controlled Oral Word Association; JOLO, Judgement of Line Orientation.

The remaining NIH ToolBox cognitive tests not used in the construction of *g* were used to compare TLE and control groups and ascertain the impact of covarying *g* in those comparisons (Fig. 1C). Without adjustment, processing speed (Pattern Completion Processing Speed) and word knowledge (Vocabulary) were significantly different between groups. After adjusting for *g*, only processing speed remained significant.

#### Clinical seizure and sociodemographic correlates of *g* (Aim 3)

**Figure 2:**
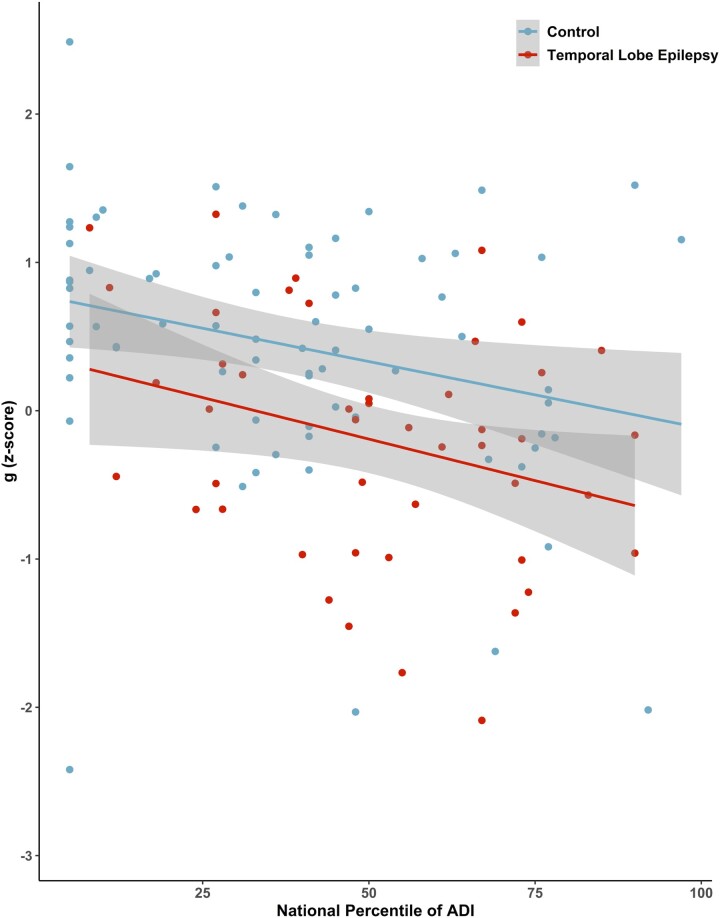
**Association of *g* with ADI.** Student's *t*-test was performed for controls (*n* = 79) (*t*_1,187_=−2.401, *P* = 0.02) and TLE participants (*n* = 110) (*t*_1,187_=−2.134, *P* = 0.038) to evaluate the significant relationship between decreasing neighbourhood disadvantage and increasing *g*. ADI, Area Disadvantage Index; TLE, temporal lobe epilepsy.

Significant associations of *g* with clinical seizure features demonstrated lower *g* with earlier age of medication onset (*r* = −0.250, *P* = 0.009), earlier age of onset of recurrent seizures (*r* = −0.187, *P* = 0.05), increasing number of antiseizure medications (*r* = −0.25, *P* = 0.01), longer duration of epilepsy (*r* = −0.196, *P* = 0.04) and increasing number of estimated lifetime generalized tonic–clonic seizures (*r* = −0.38, *P* < 0.001). There was no significant association of *g* with age of onset of first seizure (*r* = −0.178, *P* = 0.06). Sociodemographic correlates included higher *g* with increasing years of education for mother (*r* = 0.217, *P* = 0.027), father (*r* = 0.38, *P* < 0.001) and patient (*r* = 0.46, *P* < 0.001), as well as lower neighbourhood deprivation (*r* = 0.311, *P* = <0.001). Figure 2 shows the significant relationship between decreasing neighbourhood disadvantage and increasing *g* in both the TLE and controls groups.

### Neuroimaging

#### Association of *g* with structural and functional measures (Aim 4)

##### Volumetric correlates of *g*

Amongst TLE participants, increasing *g* was significantly correlated with increasing total age-adjusted ICV (*r* = 0.286, *P* = 0.019) and age and ICV-adjusted total tissue volume (*r* = 0.24, *P* = 0.19). Examination of *g* with age and ICV-adjusted tissue types revealed significantly increased *g* in association with increasing left (*r* = 0.20, *P* = 0.047) with a trend for right hemisphere cortical white matter (*r* = −0.17, *P* = 0.099). Left and right hemisphere grey matter was not associated with *g* (both *P*s > 0.17). Age- and ICV-adjusted total corpus callosum volume was significantly associated with increasing *g* (*r* = 0.258, *P* = 0.012) and examination of callosal subregions demonstrated significantly increasing *g* with increasing volumes of the posterior (*r* = 0.30, *P* = 0.003), mid-posterior (*r* = 0.232, *r* = 0.023) and anterior (*r* = 0.235, *P* = 0.022) regions, but not with central or mid anterior segments (*P* > 0.14). There was no significant association of *g* with volumes of hippocampus or thalamus (all *P*s > 0.05).

##### Cortical surface-based analyses of *g*

**Figure 3:**
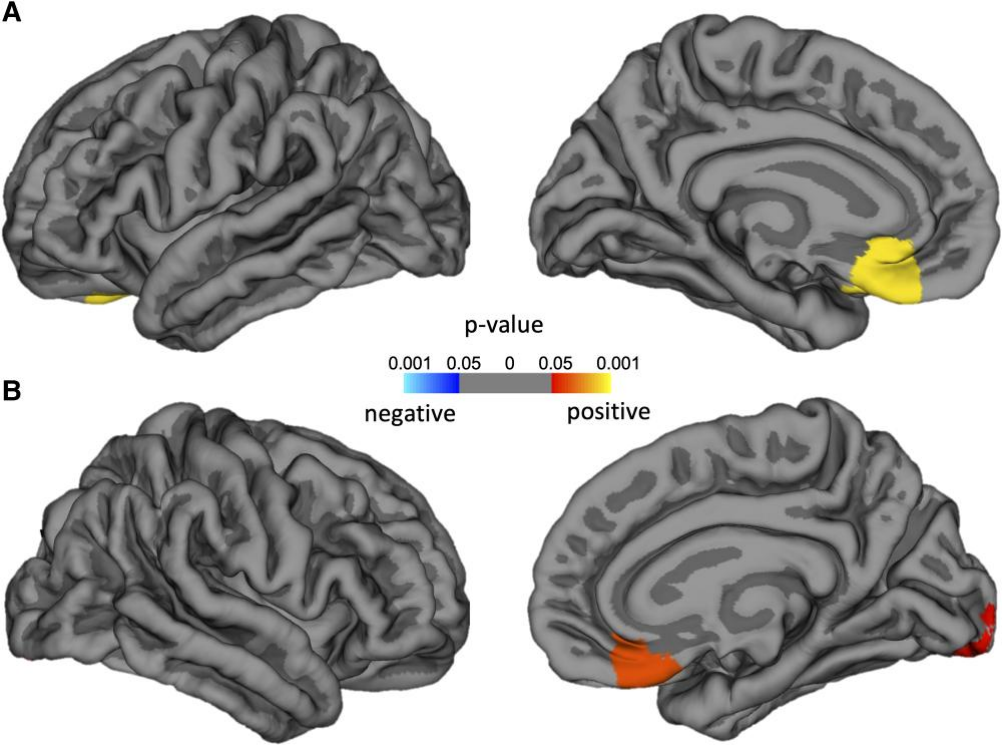
***g* score correlation with Local Gyrification Index on TLE (*n* = 101) controlling for age.** (**A**) Left hemisphere. (**B**) Right hemisphere. Significant clusters of correlation were found in bilateral medial orbitofrontal and right lateral occipital gyrus. To correct for multiple comparisons, a Monte Carlo simulation was implemented with cluster-forming threshold set to *P* = 0.05.

There was no association of *g* with FreeSurfer surface-based analyses of cortical volume, thickness or area, but significant relationships were detected between *g* and increasing local gyrification in the bilateral medial orbitofrontal regions in the epilepsy (see Fig. 3). There were no significant relationships between Local Gyrification Index and *g* in the control group.

##### GT analyses and correlations of *g* with resting-state MRI

**Figure 4:**
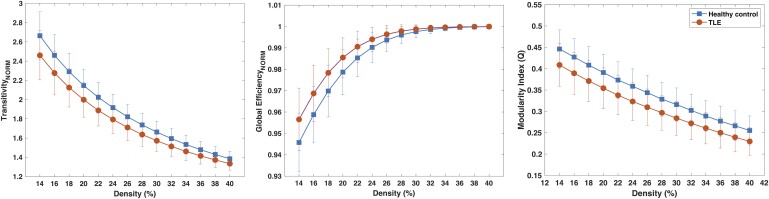
**Global GT measures.** Normalized transitivity (left), normalized global efficiency (middle) and modularity index (*Q*) (right) in healthy controls (squares) (*n* = 50) and TLE participants (circles) (*n* = 101) across density levels. Student's *t*-test was performed; results are significantly different at each density level for normalized transitivity and *Q* (*t*_1,149_ > 4, *P* < 0.001) and for density levels from 14 to 26% for normalized global efficiency after Bonferroni correction (*t*_1,149_ < −4, *P* < 0.001). TLE, temporal lobe epilepsy.

Normalized global clustering (i.e. transitivity), normalized global efficiency and modularity index (*Q*) were calculated for each participant, and averages across density levels are depicted in Fig. 4. TLE participants were below controls on global clustering and modularity indices; however, they portrayed higher global efficiency across density levels. These results suggest that in TLE there is a loss of small-world architecture and modularity with low intercommunication in specialized nodes in TLE. The greater normalized efficiency may suggest a higher transfer of information in TLE but could be counterproductive if related to increased synchrony within the epileptic network and not normal cognitive processing.

**Figure 5:**
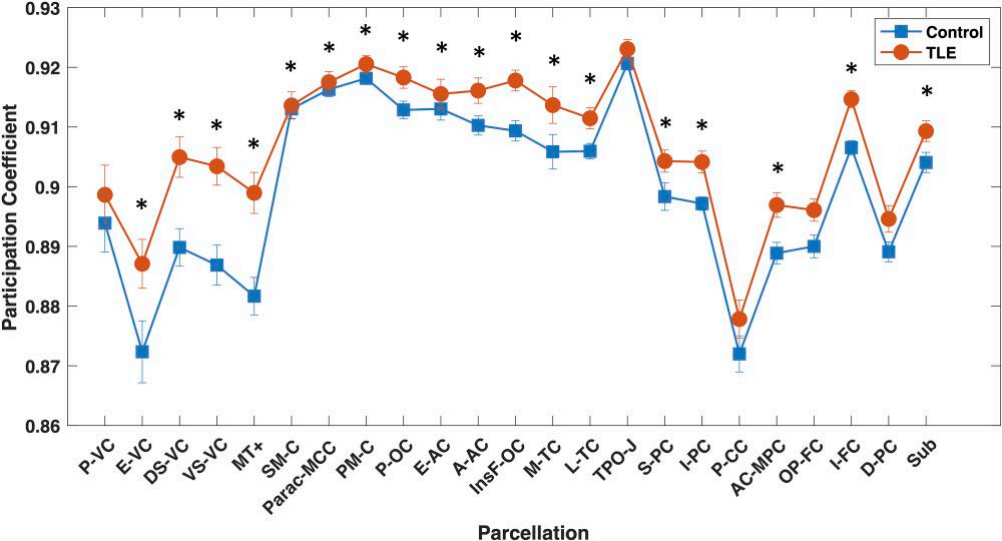
**Average PC across parcellations.** Controls (*n* = 50) are the squares and temporal lobe epilepsy participants (*n* = 101) are the circles. ANOVA was performed between groups; *significantly different between groups after false discovery rate correction (*F*_1,149_ > 5, *P* < 0.05). Abbreviations can be found in Supplementary Table 1. TLE, temporal lobe epilepsy.

In addition, the PC was calculated based on 23 parcellations,^52^ which are networks of nodes that should share similar cortical architecture, function, connectivity and topography. It was calculated for each participant and non-parametric (i.e. Spearman) correlations were calculated between each one and *g*. Figure 5 shows the average PC for each parcellation between TLE and controls, in which TLE participants present higher values than controls for each, 18 of them retaining significance after false discovery rate correction, consistently significant across density values (Supplementary Figs. 1 and 2). Therefore, nodes within Glasser parcellations in TLE participants appear to be connecting mainly with nodes from other parcellations, whilst parcellations in controls mainly connect within parcellations, especially for those 18 regions. Given that the modularity index was lower in TLE, this group of patients has low intermodular connections in general. Altogether, this adds to the disadvantageous impact on specialized functional processes in TLE participants.

Lastly, PC for each parcellation was correlated with *g* in order to explore more thoroughly if and how higher PC in TLE might be affecting cognition. Indeed, we found that TLE presented five parcellations with *g* correlations of *P* < 0.05: early visual cortex (E_VC) (*r* = 0.209, *P* = 0.043), ventral stream visual cortex (VS_VC) (*r* = 0.233, *P* = 0.024), posterior opercular cortex (*P*_OC) (*r* = 0.221, *P* = 0.032), medial temporal cortex (MT_C) (*r* = 0.245, *P* = 0.017) and superior parietal cortex (S_PC) (*r* = 0.321, *P* = 0.002). Of these, only S_PC remained significant after false discovery rate correction, which was a consistent result for a density of 20%. No significant correlations with *g* were found in controls.

## Discussion

This investigation to the best of our knowledge represents the first focused examination of a general ability metric, so-called Spearman's *g*, in epilepsy. Four aspects of this investigation consistent with the study aims are addressed below.

### *g* in TLE and control groups (Aim 1)

From a comprehensive neuropsychological test battery, a metric reflective of *g* was identified and found to be significantly lower in epilepsy compared with healthy control participants. As noted, there has been a long history of interest in the relationship between epilepsy and intelligence, dating to 1912, encompassing a wide variety of tests of intelligence in children, adolescents and adults with epilepsy that have varied widely in their content, theory and intent. The focus of the neuropsychology of epilepsy has shifted to a large degree from metrics of intelligence to tests of specific cognitive abilities (e.g. memory, language and executive function). But for unknown reasons, the very well-established general ability factor (*g*) has never been a focus of interest in epilepsy, unusual given the broad interest in *g* in the field of individual differences and intelligence and the prominent role of *g* in models of human cognition. But *g* is clearly present and impacted in epilepsy compared with controls.

### Explanatory power of *g* and domain-specific tests (Aim 2)

We next addressed the influence of *g* compared with specific cognitive abilities when making epilepsy versus control group comparisons, followed by determination of the unique variance in specific cognitive abilities after controlling for *g*'s contribution. In typical clinical and research settings, intelligence tests are often used as a descriptive measure of an individual or cohort, with primary interest in the domain-specific cognitive findings. The impact of intelligence or general ability on the domain specific measures has not been fully appreciated, although suspected to attenuate group differences on domain specific tests.^9^ This was addressed here in two ways. The comparative effect size of *g* compared with the traditional measures in the epilepsy versus control comparisons demonstrated that *g*'s effect size was larger than any individual test metric speaking to its general explanatory power. Additionally, the machine learning technique of LDA was used to create a single latent variable composed of a weighted linear combination of cognitive tests to discriminate the TLE and control groups. This variable was highly correlated with *g* at 0.84, more than any individual test. The next closest metric was total verbal learning (Rey auditory verbal learning test) at 0.79. So, *g* appeared better at predicting whether a subject was control versus TLE participant than any single cognitive test and is arguably comparable with machine learning methods applied to discriminate groups.

To begin to address the question of the unique variance in specific cognitive metrics after controlling for *g*'s contribution in epilepsy versus control comparisons, our focus turned to test measures not involved in the construction of *g*. These independent metrics derive from the NIH Tool Box Cognitive Battery and were administered during the same assessment session. The results showed that only one NIH Toolbox measure provided information independent of *g*, a metric of processing speed.

This subanalysis and examination of effect sizes (Table 2) provide some suggestion of the influence of general mental ability when making group comparisons as well as the unique role and independence (from *g*) of processing speed in epilepsy. Cognitive and psychomotor processing speed, reflected across diverse speed-based test measures, has revealed prevalent slowing in children and adults with epilepsy and, whilst related to medication treatment,^62-65^ slowing has been observed in drug naïve new-onset paediatric and adult patients with epilepsy,^66-68^ implicating causative factors other than medications. Slowing of processing speed is amongst the most prominent cognitive declines over the progressive course of both new onset and chronic epilepsy.^69,70^ Slowing remains evident even after epilepsy remits spontaneously or following treatments such as epilepsy surgery.^71-73^ Application of machine learning analytics has shown slowed processing speed to be the cognitive ability with the most power to discriminate patients with epilepsy from controls and to be associated with underlying anomalies in brain structure and connectivity.^32^ Importantly, slowing mediates performance across other diverse cognitive measures in epilepsy and its influence is magnified in the content of increasing task difficulty.^74,75^ In prospective studies of youth with new-onset epilepsies, slowing of baseline processing speed predicts an increased risk of behaviour problems 3 years later,^76^ linked to abnormal underlying cortical and subcortical structural features,^77^ and demonstrated to be associated with abnormal development of large-scale neural networks over a 2-year course.^78^ Slowing, as measures in numerous ways, appears to have a unique role in the neuropsychology of epilepsy including its independence from *g*.

### Clinical and sociodemographic predictors of *g* (Aim 3)

Moving to relate *g* to clinical epilepsy and sociodemographic factors, issues related to the concurrent validity of *g* are important to investigate. In a univariate model, *g* was significantly inversely associated age at first seizure, age at diagnosis and number of antiseizure medications—factors that have been shown to have effect in the general epilepsy literature across domain-specific measures. In a multivariate model, several factors were associated with *g* including parental years of education, years of patient education and inversely with the number of estimated lifetime secondarily generalized seizures, but not seizure laterality, age, gender or race. Neighbourhood deprivation was also related to *g* in that higher *g* was associated with less disadvantage, consistent with recent investigations of adults and children with epilepsy where greater disadvantage was associated with poorer cognition.^79,80^ In fact, in a model using *g* as the dependent variable with both national ADI percentile group as independent variables, both remain significant (ADI: *P* = 0.0018 and group: *P* = 0.00118) indicating independent effects.

### Neuroimaging of *g*: volumes, cortical surface features and resting-state networks (Aim 4)

Finally, the structural and functional imaging correlates of increasing *g* in epilepsy were larger head size (estimated total ICV) confirmed by greater total cerebral tissue volume, increased hemisphere connectivity (increasing volumes of the anterior, mid-posterior and posterior corpus callosum) and increasing gyrification in the bilateral middle orbitofrontal regions.

As expected, the global GT metrics (modularity, global efficiency and transitivity) demonstrate a disruption of the expected modular functional network architecture of the brain in patients with TLE. Expanding on this finding, we examined the PC within the network parcellation scheme from the Human Connectome Project and found that TLE patients have more than expected involvement of regions outside of the networks. When examined in association with *g*, several networks were implicated as being disrupted, early visual processing, ventral visual processing (temporal visual association regions), posterior opercular region and mesial temporal and most significantly the superior parietal. After correction for multiple comparisons, the superior parietal network remained. No correlations with *g* were found within the control group. These findings could be interpreted as TLE exerting dysfunction not only in mesial temporal lobe structures but also in regions associated with higher-level information processing that occurs in the posterior temporal/opercular regions and, in particular, the superior parietal association areas. One interpretation of these results is that in contrast to a passive structural lesion like a focal stroke, the epileptic network is not quiescent. Even in the interictal state the epileptic network disrupts information processing at the highest levels and why the *g* factor has a large effect size and subsumes much of the variance from other cognitive domains.

### Limitations and future directions

This investigation has imitations. The TLE participants did not uniformly undergo ictal monitoring, which prevented an unequivocal assessment of laterality effects. The number and range of tests independent from the construction of *g* was limited, and future research is needed to more clearly assess the independence of domain-specific cognitive metrics from *g* in epilepsy–control comparisons. *g* needs to be examined in other epilepsies in children and adults with epilepsy and its clinical utility interrogated.

## Conclusion

The tradition in clinical neuropsychology, and the neuropsychology of epilepsy in particular, has been a differentiation of cognitive abilities via domain-specific assessments. A focus on a single general cognitive metric is antithetical to contemporary practice and theory. But diverse cognitive and domain-specific metrics are intercorrelated (so-called positive manifold). The neuropsychology of epilepsy has yet to fully come to terms regarding how best to consider measures of general ability when examining specific abilities such as memory or executive function. When covaried, differences between epilepsy and control groups can be attenuated and variance lessened.

In conclusion, Spearman's *g* is present in TLE and is linked to *brain* (size, connectivity and regions), *environment* (family education, neighbourhood deprivation) and *disease* (onset, treatment and duration). Greater attention to general models of human cognition will lead to additional insights concerning the neuropsychology of epilepsy.

## Supplementary Material

fcae176_Supplementary_Data

## Data Availability

Efforts are ongoing to release raw DICOM (digital imaging and communications in medicine) data from the Epilepsy Connectome Project through the CCF (Connectome coordination facility: www.humanconnectome.org/software/connectomedb; Hodge *et al*.^81^) at Washington University in St. Louis.
