# Supplementary material for: The relevance of Spearman's g for epilepsy
Source: Brain Commun. 2024 Jun 12;6(3):fcae176. doi: 10.1093/braincomms/fcae176 (PMC11179110; doi:10.1093/braincomms/fcae176)
Supplement: fcae176_Supplementary_Data [file fcae176_supplementary_data.pdf]

Supplementary Material

I. Supplementary Table 1

	Network	Abbreviation
1	Primary Visual Cortex (V1)	P_VC
2	Early Visual Cortex	E_VC
3	Dorsal Stream Visual Cortex	DS_VC
4	Ventral Stream Visual Cortex	VS_VC
5	MT+ Complex and Neighboring Visual Areas	MT+
6	Somatosensory and Motor Cortex	SM_C
7	Paracentral Labular and Mid Cingulate Cortex	Parac_MCC
8	Premotor Cortex	PM_C
9	Posterior Opercular Cortex	P_OC
10	Early Auditory Cortex	E_AC
11	Auditory Association Cortex	A_AC
12	Insular and Frontal Opercular Cortex	InsF_OC
13	Medial Temporal Cortex	M_TC
14	Lateral Temporal Cortex	L_TC
15	Temporo-Parieto-Occipital Junction	TPO_J
16	Superior Parietal Cortex	S_PC
17	Inferior Parietal Cortex	I_PC
18	Posterior Cingulate Cortex	P_CC
19	Anterior Cingulate and Medial Prefrontal Cortex	AC_MPC
20	Orbital and Polar Frontal Cortex	OP_FC
21	Inferior Frontal Cortex	I_FC
22	DorsoLateral Prefrontal Cortex	D_PC
23	Subcorticals	Sub

II. Participation coefficient across parcellations for different density levels

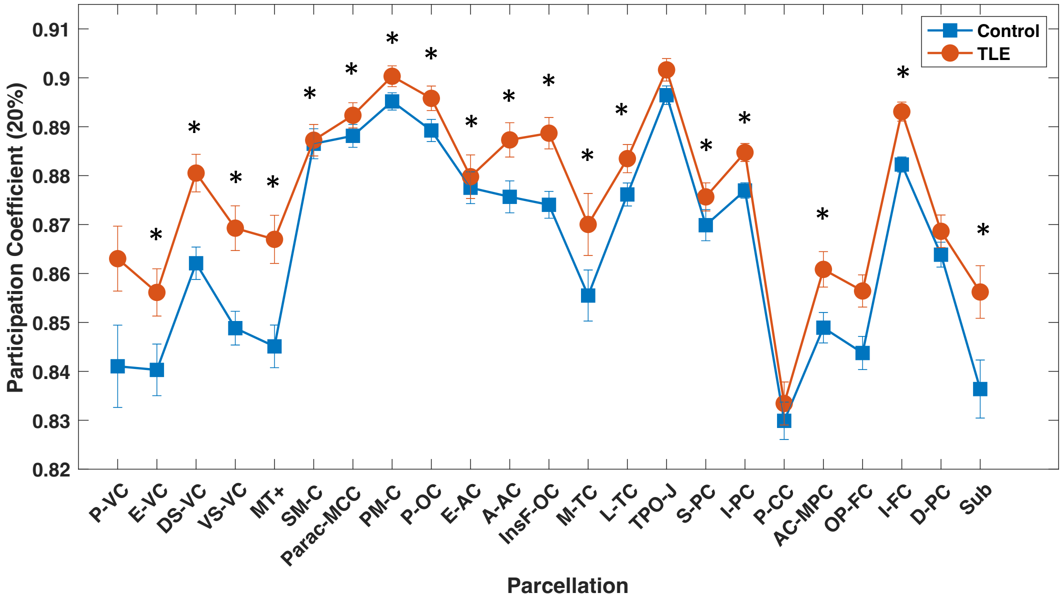

Supplementary Figure 1: Participation coefficient across parcellations for TLE (red) (n=101) and controls (blue) (n=50) at a density of 20%. Error bars represent the standard deviation. ANOVA was performed between groups; *significantly different between groups after FDR correction ($F_{1,149}$>5, p<0.05).

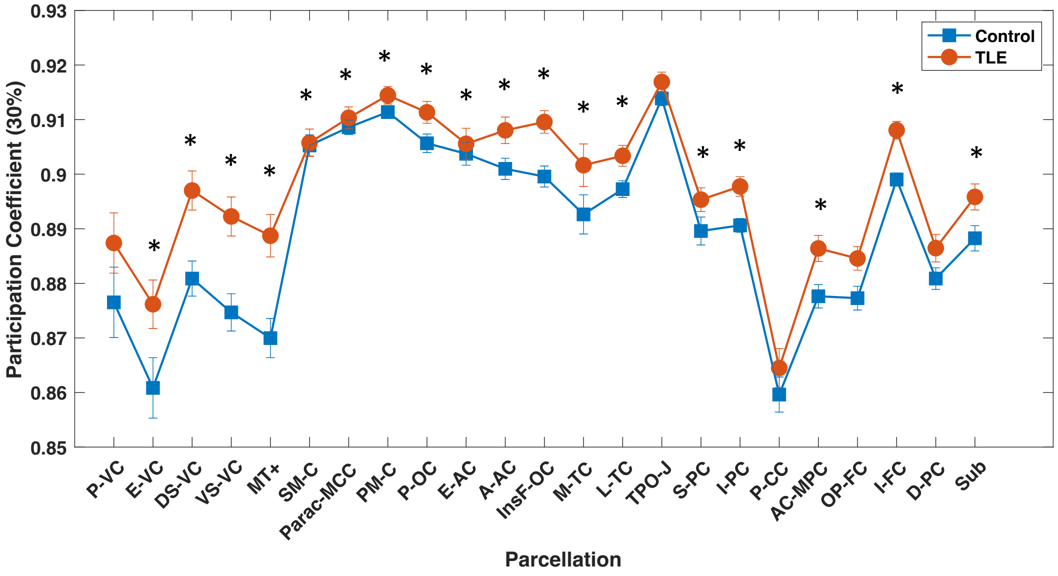

Supplementary Figure 2: Participation coefficient across parcellations for TLE (red) (n=101) and controls (blue) (n=50) at a density of 30%. Error bars represent the standard deviation. ANOVA was performed between groups; *significantly different between groups after FDR correction ($F_{1,149}$>5, p<0.05).

III. Comparisons of distributions of “g”

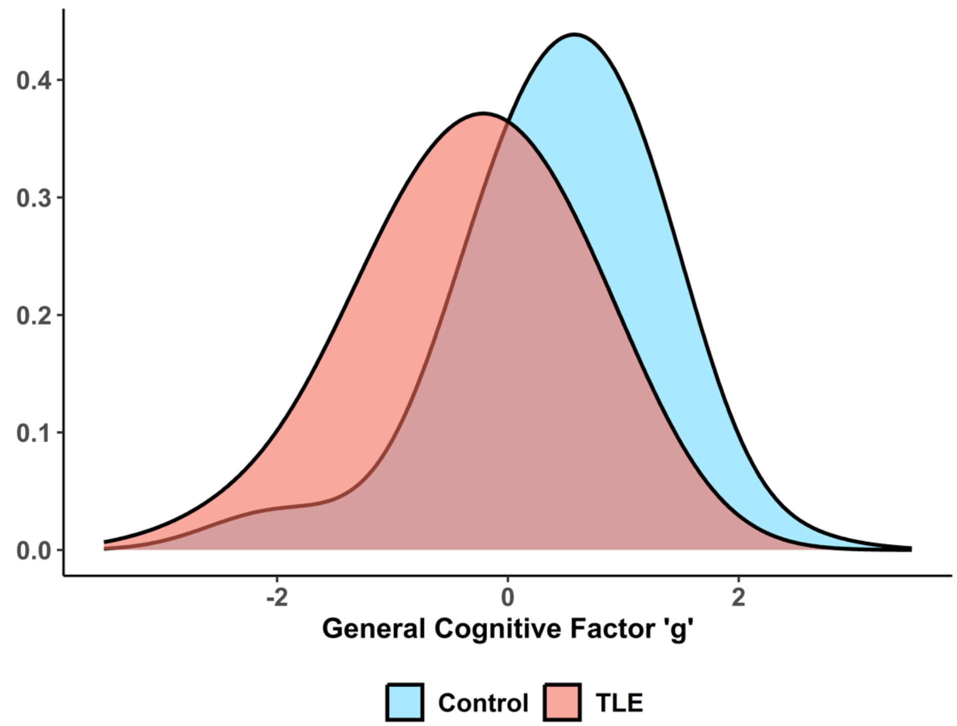

Supplementary Figure 3: Comparisons of distributions of g for controls (blue) and temporal lobe epilepsy (TLE) participants (red).
